# Visceral Adipose Tissue Influence on Health Problem Development and Its Relationship with Serum Biochemical Parameters in Middle-Aged and Older Adults: A Literature Review

**DOI:** 10.1155/2022/8350527

**Published:** 2022-04-19

**Authors:** Vanessa C. Moreira, Calliandra M. S. Silva, Alexis F. Welker, Izabel C. R. da Silva

**Affiliations:** Health Sciences and Technologies, University of Brasilia, Zip-Code: 72220-275, Brasilia, Brazil

## Abstract

**Background:**

The amount of visceral adipose tissue (VAT) tends to increase with age and is associated with several health problems, such as cardiometabolic diseases, increased infections, and overall mortality.

**Objectives:**

This review provides a general assessment of how visceral adiposity correlates with the development of health problems and changes in serum biochemical parameters in middle-aged and older adults.

**Methods:**

We searched specific terms in the Virtual Health Library (VHL) databases for VAT articles published in the English language between 2009 and 2019 related to older adults.

**Results:**

The search found twenty-three publications in this period, of which nine were excluded. The publications had a population aged between 42 and 83 years and correlated the VAT area ratio with several comorbidities (such as pancreatitis, depression, cancer, and coronary heart disease) and serum biochemical parameters.

**Conclusion:**

Further research on the association between visceral obesity and the emergence of health problems and the relationship between VAT and changes in serum biochemical parameters in older individuals should deepen the understanding of this connection and develop preventive actions.

## 1. Introduction

Obesity has been one of the rising comorbidities globally, reaching pandemic proportions to become the foremost cause of preventable death. This condition is associated with a decreased life expectancy of about 5 to 20 years, depending on the severity and other associated comorbidities [1]. According to the World Health Organization (WHO), an individual is classified as obese if their body mass index (BMI) is greater than or equal to 30 kg/m^2^. Individuals with a BMI between 25 and 29.9 kg/m^2^ are classified as overweight and may already present health risks from excess fat [2].

The population's life expectancy has increased in recent years compared with the past decade, and although obesity in young people is a risk factor for morbidity and mortality, the effect of obesity in older adults is much more complicated [3]. Studies show that when analyzing the link between age and body fat distribution, overweight and obesity increase with age up to about 70 years, and afterward, it tends to decrease [4, 5]. Aging is associated with body fat distribution changes mainly due to visceral adipose tissue (VAT) accumulation, known as visceral obesity [6].

Visceral obesity is characterized as increased adipose tissue (fat) around the intra-abdominal organs and can also be called abdominal or central obesity [7]. Quantitative and qualitative VAT analysis can be determined by gold standard diagnostic techniques such as dual-energy X-ray absorptiometry (DXA, formerly DEXA), computed tomography, magnetic resonance, ultrasonography, or a combination of techniques. Anthropometric standards such as waist circumference and waist-to-hip ratio, for example, can also assess the presence of central obesity, but not its quantity and quality [7, 8].

Excessive deposition of VAT influences the pro-inflammatory response regulation, and with aging, adipocytes tend to have a higher pro-inflammatory level due to adipokines' action [9]. Thus, an elevated VAT area contributes to a greater risk of metabolic and hemodynamic disorders, favoring the appearance of diseases, such as arterial hypertension, diabetes, atherogenic cardiovascular disease, coronary disease, and kidney problems [10], and changes in serum biochemical parameters [11, 12].

In addition to obesity, another relevant factor for older adults' health is the decline in skeletal muscle mass associated with functional deterioration, known as sarcopenia. This condition leads to higher rates of disability and frailty and has been associated with more extended hospital stays, increased infections and postoperative complications, increased chemotherapy toxicity, and worse recurrence-free survival and overall survival [13]. In the context of progressive population aging, the association of obesity, particularly VAT, with loss of lean mass leads to sarcopenic obesity, which is independently associated with increased mortality, cardiovascular complications, and cancer progression [14]. The VAT accumulation and the relative muscle mass loss analysis can become, in old age, an essential component in determining the health risk associated with obesity [14].

The number of studies on visceral obesity epidemiology in middle-aged and older adults, mainly in developing countries, is still limited. The available studies reveal an increase in this condition's prevalence in older individuals and that visceral adiposity should be considered a severe health epidemic worldwide, which reaffirms the need to expand knowledge about central obesity in this population group. In this context, this review aims to assess the correlation between visceral adiposity and the development of health problems and possible changes in serum biochemical parameters in middle-aged and older adults.

## 2. Materials and Methods

### 2.1. Search Strategy

We performed a bibliographic search through the Virtual Health Library (VHL) database. This bibliographic database includes publications found in LILACS, MEDLINE, and other information bases, such as open educational resources, websites, and scientific events. The search took place in January 2020, researching publications that show information on visceral adipose tissue (VAT) area analysis in older adults. The search terms used were as follows: “visceral adipose tissue area” and “elderly.” A hierarchical approach was employed to assess the studies' relevance based on their title, summary, and full report. The Preferred Reporting Items for Systematic reviews and Meta-Analyses (PRISMA) checklist was used to analyze and report the search results [15].

### 2.2. Study Selection

The filters used were related to full-text open access, database, language, and year of publication. The literature search was unrestricted regarding the species and study type, and two reviewers (V. M. and I. S.) independently evaluated the identified articles. The inclusion criteria were as follows: (1) the study should address the VAT area in older adults, regardless of the diagnostic methodology employed; (2) the study should correlate VAT with comorbidities or serum biochemical tests; (3) the study must be published in the MEDLINE database; (4) the study must be in English; and (5) the study must have been published in the past ten years (2009 to 2019). We excluded studies if they had the following: (1) opinion or review; (2) only address muscle mass; (3) related the drug or supplement effect on VAT; (4) intended for validation of a novel VAT area estimator; and (5) studies to determine VAT by analyzing imaging tests, programs, and software protocols.

### 2.3. Data Extraction and Analysis

All identified articles were screened independently by two authors (V. M. and I. S.) based on title and abstract for inclusion or exclusion, and all those that met the inclusion criteria had their full text also independently assessed by V. M. and I. S. Any discrepancies in the inclusion and exclusion criteria assessment were discussed until a consensus was reached.

V. M. and I. S. independently reviewed eligible articles and extracted relevant data. The gathered information included authors' names, study title, objective, year of publication, the country where the study took place, sample size, population's age group, study type, analyzed comorbidity, laboratory tests associated with VAT, research's results, and statistical values.

## 3. Results

The keyword search identified 23 articles from MEDLINE. After subjecting the articles to title and abstract review, we excluded three articles for not meeting inclusion criteria: software programs and protocols for imaging examination studies, respectively [16, 17], and one focused on muscle mass analysis [18]. After the title and abstract review, we selected 20 articles for full-text review. Of these, two were excluded for focusing on drugs or supplement effect on visceral adipose tissue (VAT) [19, 20], two for not addressing VAT association with health problems or biochemical tests [21, 22], an article that limited their comparison between the VAT estimated by bioelectrical impedance analysis (BIA) and computed tomography (CT) [8], and one intended to validate a novel VAT area estimator in Japanese people [23].

**Figure 1:**
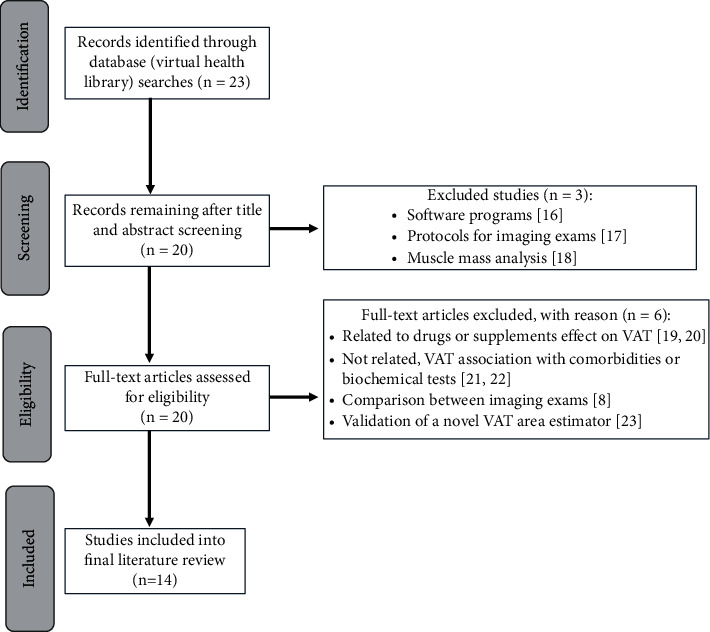
Flow chart for identification, screening, and selection of the literature.

The research did not result in duplicate articles or different studies on the same group of participants. This search selected fourteen articles published between 2009 and 2019 that met inclusion criteria and were deemed eligible to be included within this systematic review.  Figure 1 presents a flowchart with the results of the literature search process.

Of the selected articles, four were published in Japan, two in Korea, two in the Netherlands and the United States, followed by Brazil, China, Iran, and Turkey, and distributed in seven cross-sectional studies, four cohort studies, one retrospective case-control study, and one observational-retrospective study at a single center. The publications had a sample population with ages ranging from 42 to 83 years with older adults' predominance (Table 1).

### 3.1. VAT Area and Its Correlation with Health Problems and Biochemical Parameters

Of the 14 articles analyzed, seven presented an association between VAT and disease manifestations such as ischemic colitis [24], nonalcoholic fatty hepatitis [25], acute pancreatitis [26], depression [27], and coronary artery disease [28].

Three studies related visceral adiposity to procedures' outcomes, such as cecal insertion in colorectal cancer screening, retropubic prostatectomy, and renal function after nephrectomy in living donors [29–31]. VAT correlation with muscle mass was addressed by three studies: two related to cancer-related sarcopenia [32, 33] and one related to sarcopenic obesity [34].

As for serum biochemical tests, only three studies showed a direct association between VAT patterns and serum levels of uric acid, amylase, and lipid profile [11, 12, 35].

## 4. Discussion

Our literature review presented visceral adipose tissue (VAT) association with several health problem manifestations—such as colitis, cancer, pancreatitis, depression, and coronary and liver diseases—in middle-aged and older adults.

Studies indicate that VAT closely correlates with nonalcoholic fatty liver disease (NAFLD) and risk factors for coronary artery disease such as dyslipidemia, diabetes, and metabolic syndrome [36, 37]. In his study, Kim et al. [25] observed that individuals with NAFLD have a high risk of coronary atherosclerosis, regardless of traditional cardiovascular risk factors, such as visceral adiposity. VAT attenuated, but did not eliminate, the relation between NAFLD and artery calcification, suggesting that NAFLD's presence may be an independent risk factor for coronary artery disease.

NAFLD association with atherosclerosis may be related to the hepatic pro-inflammatory process that stimulates reactive oxygen species to induce cytokine production (such as tumor necrosis factor-alpha (TNF-a) and interleukin-6 (IL-6)) that, together with increased hepatic C-reactive protein levels, add more atherogenic stimuli to the body [38]. Furthermore, low adiponectin serum levels, which are inversely related to NAFLD severity, play a vital role in the pathogenesis between NAFLD and subclinical coronary atherosclerosis [37] since it regulates the inflammatory response by inhibiting macrophages and cytokine production, impacting the diseases' manifestation and severity [9].

Visceral adiposity correlates with an increased likelihood of having coronary artery disease, and if the disease is already present, it will be more diffuse compared with patients without visceral adiposity [39, 40]. Ohashi et al. [28] also reported the VAT area's association with coronary artery calcium (CAC)'s presence and extension, regardless of body mass index (BMI). Increased VAT area likewise correlated significantly with noncalcified coronary plaques' presence and extent, contributing to atherosclerosis's acceleration, regardless of the traditional cardiovascular risk factors, such as hypertension, hypercholesterolemia (high cholesterol levels in the bloodstream), and diabetes or other body composition parameters, such as the subcutaneous adipose tissue (SAT) area, BMI, and waist circumference (WC) [28]. Such findings may explain the excessive cardiovascular risk in patients with visceral adiposity.

Coronary artery calcification correlated with cardiac events' risk, indicating coronary atherosclerosis presence and extension [41]. In addition to pro-inflammatory cytokine action and increased plasminogen activator-1 levels, the presence of insulin resistance (a common condition in visceral obesity) promoted by free fatty acids increases atherosclerotic plaque's atherogenesis and instability, further increasing the tissue's inflammatory process [42]. Such findings may explain the excessive cardiovascular risk in patients with visceral adiposity and improve risk stratification, helping to identify viscerally obese patients at high risk of cardiovascular events and apply lifestyle modification strategies and pharmacological interventions [28].

Low adiponectin levels and increased lipotoxicity present in individuals with visceral obesity may also explain the relationship between VAT and acute pancreatitis manifestation [9]. Natu et al. [26] noted that the VAT area was significantly larger in individuals with severe pancreatitis than in those with mild or moderate disease. Those with an increased VAT area had a higher incidence of persistent systemic inflammatory response syndrome, acute necrotic collections, and multisystem organ failure. With each 25 cm^2^ increase in the VAT area, the probability of a severe episode rose by 20%. These findings suggest that an increase in the VAT area is a strong predictor of severe pancreatitis, necrosis, systemic inflammatory response syndrome, and multisystem organ failure.

VAT accumulation is also associated with outpatient ischemic colitis development. According to Aoki et al. [24], even when BMI was considered simultaneously, colitis remained associated with abdominal obesity, suggesting that the primary mechanisms for developing ischemic colitis are atherosclerosis and inflammation potentiated by VAT accumulation. As previously discussed, VAT tends to stimulate the release of cytokines (such as IL-6 and TNF-a) and hormones that can lead to systemic inflammation, thereby increasing coagulation and adhesion molecules, contributing to atherosclerosis formation [43]. Visceral obesity also correlates with metabolic syndrome—characterized by hypertension, diabetes mellitus, and dyslipidemia, all risk factors that also contribute to atherosclerosis—and increases plasminogen activator inhibitor-1 levels, making abdominal visceral fat a potential risk factor for mesenteric artery thrombosis [44].

Another association found in this systematic review was between depression and VAT accumulation. Depressive symptoms correlated with intra-abdominal fat and women's proportion of visceral and total adipose tissue area after controlling for confounding factors, such as BMI, hypertension, and diabetes. In men, diabetes mellitus presence and high BMI significantly correlated with depressive symptoms. According to the study, for every 1 cm^2^ of VAT area in women, the risk of individual inclusion in the clinically depressed group increases by 1.006 times, whereas for a 1% increase in the visceral and total adipose tissue area ratio, the risk of being clinically depressed increases 1.028 times [27]. Some hypotheses might explain the connection between VAT and depressive symptoms, such as the hormone cortisol action [45], the hypothalamic-pituitary-adrenal axis deregulation [46], the inflammatory process originating from adipose tissue [47], and insulin resistance, common in individuals with high VAT [48]. High triglyceride levels, low HDL-c (low-density lipoprotein) levels, high blood pressure, and high glucose levels can also contribute in some way to the association of VAT with depression [49].

Another three articles analyzed correlated VAT with the outcome of invasive procedures such as nephrectomy, radical retropubic prostatectomy, and cecal insertion. When analyzing individuals that underwent colonoscopy for colorectal cancer detection/monitoring, Nagata et al. [30] observed that VAT's relative distribution might also have different effects on the risk of more prolonged cecal insertion time. However, the excessive SAT accumulation has demonstrated a better predictive factor for easier colonoscope passage, even in patients with average weight. A possible explanation is that a larger SAT area may provide resistance and help prevent sigmoid looping [50].

Obesity also significantly affects the performance of radical retropubic prostatectomy, which may be associated with the severity of post-prostatectomy incontinence and favorable surgical margin rates [51]. In this context, Ongun et al. [31] observed that an elevated VAT area might impact the trifecta failure in individuals undergoing radical retropubic prostatectomy, as well as the presence of positive surgical margins, higher prostate-specific antigen (PSA) levels, lower prostate volume, and narrower prostate width in older individuals. These findings suggest that the pelvic and VAT biometric measurements may help preoperative planning and prostatectomy management.

Obesity can be an independent risk factor for developing chronic kidney disease (CKD) and hypertension [52]. According to Hori et al. [29], the preoperative VAT distribution and nutritional status may predict postoperative renal function in living donors more than the BMI. Moreover, donors with preoperative hypertension tend to have a VAT area ≥80 cm^2^, hinting at obesity being a risk factor for hypertension. Hence, living donors' preoperative management regarding visceral obesity, hypertension, and nutritional status may lead to better results and help maintain renal function after donor nephrectomy.

Among the studies analyzed, three articles showed an association between muscle mass and visceral adiposity. The primary approach was toward sarcopenia, a clinical condition resulting from the involuntary loss of skeletal muscle with or without losing body fat from a disease [53]. Sarcopenia is a common condition in individuals over 70 and patients with advanced cancer, contributing to death in almost all cancer patients by enhancing their sensitivity to chemotherapy toxicity and increasing the risk of developing postoperative complications [54, 55].

Visceral obesity and sarcopenia are relatively common in individuals diagnosed with colorectal cancer [56]. Although there is evidence that obesity is associated with worse health-related quality-of-life outcomes in colorectal cancer survivors, van Roekel et al. [33] found no significant association between visceral obesity and sarcopenia with long-term health-related quality of life in survivors of colorectal cancer in stages I to III. In contrast, Malietzis et al. [57] observed an association of these body parameters with worse clinical results and short-term survival. These results suggest that interventions targeting patients with colorectal cancer with unhealthy body composition at the time of diagnosis may be favorable for improving clinical outcomes and short-term survival but may not be essential for improving the quality of life related to long-term health [58].

The amount of visceral adipose tissue also appears to impact patients with pancreatic cancer, although sarcopenia also correlated with poor results in several other types of cancer [59]. Low attenuation of muscle radiation was associated with reduced survival after pancreatic surgery. In contrast, elevated VAT was associated with the risk of infections at the surgical site in patients with pancreatic head cancer, showing the importance of preoperative body composition analysis to reduce risks [32]. The increased risk of infection may be related to wound healing and tissue oxygenation problems, common in individuals with visceral obesity in which arterial function and angiogenesis tend to be impaired [60].

The association of obesity with sarcopenia, called sarcopenic obesity, was the only independent risk factor for 30-day mortality in critically ill patients with intra-abdominal sepsis, indicating the need for sarcopenic obesity assessment as part of the risk analysis in patients critically ill with intra-abdominal sepsis [34].

Sarcopenia and visceral obesity are considered multifactorial syndromes with various causes and interconnected feedback mechanisms acting in synergy [61]. Sarcopenia induces a decline in the basal metabolic rate and, consequently, reduces energy expenditure, leading to visceral obesity [62]. The increase in VAT secretes more pro-inflammatory cytokines and induces chronic inflammation, contributing to sarcopenia's development and progression [63].

As for biochemical parameters, body composition and adipose tissue distribution are critical conditions in assessing the link between obesity and adverse metabolic outcomes [12]. VAT is highly lipolytic and performs direct drainage to the liver, promoting an excessive flow of nonesterified fatty acids [64]. This phenomenon triggers changes such as very-low-density lipoprotein (VLDL-c) overproduction and, indirectly, low-density lipoprotein (LDL-c) overproduction, and a decrease in high-density lipoprotein (HDL-c) levels that might result in hypertriglyceridemia (high triglyceride levels in the bloodstream), hypercholesterolemia (high cholesterol levels in the bloodstream), and atherogenesis (fatty plaque formation in the arteries) [65]. VAT strongly correlates with the tests' results for triglycerides, VLDL-c, and uric acid in older adults, indicating a direct association between them [11]. In contrast, VAT has a low correlation with total cholesterol and LDL-c and an inverse correlation with HDL-c in both sexes. Compared with younger women, older women showed an increase in all biochemical parameters (glycemia, triglycerides, total cholesterol, LDL-c, VLDL-c, and uric acid), except HDL-c [11].

On the other hand, Sadeghi et al. [12] observed that in patients without coronary heart disease (CD), VAT had a better correlation with dyslipidemia, while in individuals with CD, waist circumference and waist-to-height ratio were better correlated with dyslipidemia. In individuals without CD, apolipoprotein A (apo A) had the highest levels, and VAT positively correlated with LDL-c, triglycerides, and apo B and inversely correlated with HDL-c. After adjusting the correlation between adiposity and lipid profile measures, individuals without CD showed an association of VAT with total cholesterol, LDL-c, triglycerides, and apo B and were inversely associated with HDL-c, regardless of the age of sex.

Triglycerides represent 99.0% of circulating fat and are substrates for the VLDL-c formation. Therefore, elevation in its concentrations is almost always accompanied by hypercholesterolemia. Reduced HDL-c serum levels, present in approximately 10.0% of the population, might be unable to eliminate excess cholesterol from the vascular walls, as it presents antiatherogenic and antioxidant effects. Notwithstanding, studies suggest evaluating cholesterol as an index and not in isolation; e.g., the total cholesterol/HDL-c ratio is considered a potent predictor for coronary heart disease due to a probable high atherogenic effect [11, 66].

Concerning uric acid, evidence points to it as a risk factor for hypertension, dyslipidemia, and glucose metabolism disorder, and it also may have a causal role in cardiovascular disease pathogenesis. However, its correlation with VAT has not yet been fully elucidated [67].

Recent studies have reported an association between amylase and obesity owning to the salivary amylase gene expression ability to predict obesity in humans [68]. Curiously, Dias and [35] observed that serum amylase is more strongly associated with VAT than SAT and BMI. Low amylase serum levels have also correlated with an increase in diabetes and metabolic syndrome cases [69]. A possible explanation for this is that saturated fatty acids stimulate pancreatic amylase release in a dose-dependent manner. However, excess circulating free fatty acids can inhibit serum amylase secretion stimulation due to a feedback loop in pancreatic cells [70]. Another explanation is the influence of insulin resistance since stimulation of amylase's secretion and synthesis occurs in response to insulin binding to its receptor in pancreatic acinar cells. If there is a resistance, such as a connection not occurring, it may reduce amylase secretion [71]. Excessive free fatty acids levels can also impair insulin receptor activity due to the accumulation of these fatty acids' metabolic by-products [70].

The findings, in general, confirm the importance of investigating VAT the strong correlation with serum biochemical parameters. However, biochemical tests are risk indicators for diseases but are not diagnostic and should be associated with complementary information such as medication use, comorbidities, lifestyle, and other factors associated with metabolic changes [11].

This review's main strength is that, to our knowledge, this is the first review that addresses the association of VAT with health problems and biochemical tests in an older adult population. Notwithstanding, there are some limitations to our study. For instance, our inclusion/exclusion criteria limited the articles reviewed as it excluded articles without open access, outside the scientific publication community, and published in other languages, which could change the results and generate some publication bias. Another limitation was the impossibility of conducting a meta-analysis due to the immense heterogeneity in the research projects and objectives among the selected studies.

## 5. Conclusions

Knowledge of age-related changes in body composition and fat distribution is essential to understand the association between obesity, morbidity, and mortality in a population's age group. Increased visceral adipose tissue (VAT) area is considered a risk factor for cancer onset and cardiometabolic and biochemical changes. A better understanding of how visceral obesity correlates with health problem development can contribute to the emergence of promising intervention strategies. In this context, visceral obesity must be treated as a public health problem, highlighting the need for further studies, especially for the older adult population, to provide healthy aging.

## Figures and Tables

**Table 1:** Comparison of different visceral adipose tissue (VAT) studies and pathogenesis in older adults.

Author	Title	Objective	Year	Study location	Sample	Population age (years)	Study	Comorbidities	Laboratory test associated with VAT	Results	*P* value
(van Roekel et al., 2017) [33]	Associations of adipose and muscle tissue parameters at colorectal cancer diagnosis with long-term health-related quality of life	Analyze the associations of body composition parameters in diagnosing colorectal cancer with long-term health-related quality of life, 2 to 10 years after diagnosis.	2017	Netherlands	104	64.3 ± 9.0	Cross-sectional	Health-related quality of life in individuals with colorectal cancer	—	There was no significant association between visceral obesity and sarcopenia with long-term health-related quality of life in colorectal cancer survivors in stages I to III.	*P* < 0.05

(Aoki et al., 2015) [24]	Abdominal fat accumulation, as measured by computed tomography, increases the risk of ischemic colitis: a retrospective case-control study	Evaluate the abdominal fat accumulation effect on the ischemic colitis development and related clinical outcomes.	2015	Japan	116	Cases (61.5 ± 17.3)	Retrospective case-control	Ischemic colitis	—	The abdominal fat accumulation, but not the BMI, is associated with ambulatory ischemic colitis development. Colitis clinical results did not correlate with abdominal obesity.	*P* < 0.05
						Controls (61.5 ± 16.9)					

(Nagata et al., 2014) [30]	Predictors for cecal insertion time: the impact of abdominal visceral fat measured by computed tomography	Identify the predictors of longer cecal insertion time and evaluate the effect of BMI, VAT, and SAT on this time.	2014	Japan	899	63.7 ± 14.3	Retrospective observational	Colorectal cancer screening	—	Among the obesity indices, the high accumulation of subcutaneous fat was the best predictive factor to facilitate the colonoscope's passage, even when the body weight was normal.	*P* < 0.05

(Kim et al., 2012) [25]	Nonalcoholic fatty liver disease is associated with coronary artery calcification	Investigate the relationship between NAFLD and coronary artery calcification, taking into account risk factors for coronary artery disease, such as VAT, in an apparently healthy population.	2012	Seoul (South Korea)	4023	56.9 ± 9.4	Cross-sectional	Nonalcoholic fatty liver disease and coronary artery calcification	—	NAFLD individuals are at high risk of developing coronary atherosclerosis, regardless of whether they have visceral obesity or classic coronary risk factors.	*P* < 0.001

(Hori et al., 2018) [29]	Impact of preoperative abdominal visceral adipose tissue area and nutritional status on renal function after donor nephrectomy in Japanese living donors for renal transplantation	Evaluate the effects of preoperative abdominal fat distribution and nutritional status on renal function after nephrectomy in living donors.	2018	Japan	75	47 to 64	Cohort	Kidney function after nephrectomy	—	Preoperative VAT distribution and nutritional status can predict postoperative renal function in living donors.	*P* < 0.05

(Natu et al., 2017) [26]	Visceral adiposity predicts severity of acute pancreatitis	Determine the association of visceral adiposity with severe outcomes in acute pancreatitis.	2017	Cleveland (United States)	252	52.0 ± 15.9	Historical cohort	Acute pancreatitis	—	An increased VAT area is a strong indicator of severe pancreatitis, necrosis, and multisystem organ failure.	*P* < 0.001

(van Dijk et al., 2017) [32]	Low skeletal muscle radiation attenuation and visceral adiposity are associated with overall survival and surgical site infections in patients with pancreatic cancer	Evaluate the association of adipose tissue and other body composition characteristics with postoperative survival, postoperative complications, and surgical site infections in patients with pancreatic head cancer.	2017	Netherlands	186	61 to 79	Prospective cohort	Surgical site infection in patients with pancreatic cancer	—	The low attenuation of muscle radiation is a predictor of survival after pancreatic surgery and the high visceral adiposity associated with the surgical site's risk of infections.	*P* < 0.05

(Dias et al., 2016) [35]	Association of abdominal fat with serum amylase in an older cohort: the baltimore longitudinal study of aging	Investigate serum amylase associations with diabetes and body fat measurements with a focus on abdominal obesity.	2016	United States	778	66.8 ± 13.6	Cross-sectional	Diabetes	Serum glucose; serum amylase	Serum amylase tends to be low in people with diabetes and is more strongly associated with VAT than with BMI or SAT.	*P* < 0.05

(Ongun et al., 2015) [31]	Impact of pelvic biometric measurements, visceral and subcutaneous adipose tissue areas on trifecta outcome and surgical margin status after open radical retropubic prostatectomy	Investigate the impact of pelvic biometric measurements, VAT and SAT areas, on the functional and oncological results of retropubic radical prostatectomy.	2014	Turkey	270	62.63 ± 6.03	Retrospective single center	Trifecta result (ability to reach urinary continence, sexual potential, and cancer control) after prostatectomy	—	Pelvic biometric measurements and a more prominent VAT area impact the trifecta result after radical retropubic prostatectomy.	*P* < 0.05

(Sadeghi et al., 2013) [12]	Abdominal fat distribution and serum lipids in patients with and without coronary heart disease	Investigate the association between different obesity rates, fat distribution indicators, and lipid profile in patients with stable angina, with or without coronary heart disease (CHD).	2013	Iran	123	With CHD (50.5 ± 7.6)	Cross-sectional	Coronary heart disease/stable angina	Serum total cholesterol; serum triglycerides; HDL-c; LDL-c; apolipoproteins A and B	The VAT area correlates better with dyslipidemia in patients without coronary heart disease.	*p* ≤ 0.05
						Without CHD (53.7 ± 7.6)					

(Roriz et al., 2010) [11]	Imaging assessment of visceral adipose tissue area and its correlations with metabolic alterations	Verify the VAT area influence on metabolic changes in adults and older adults.	2010	Brazil	194	Mean 72,97	Cross-sectional	—	Serum glucose level; serum total cholesterol; serum triglycerides; HDL-c; LDL-c; VLDL-c; uric acid	Most biochemical tests correlated strongly with the VAT area—considered a risk factor for metabolic changes. In older individuals, the risk of VAT area appears to be greater than in younger adults.	*P* ≤ 0.05

(Ohashi et al., 2010) [28]	Association between visceral adipose tissue area and coronary plaque morphology assessed by CT angiography	Investigate VAT association with noncalcified coronary plaques' presence, extent, and characteristics.	2010	Japan	427	67 ± 11	Cross-sectional	Noncalcified coronary plaques	—	An increased VAT area significantly associates with noncalcified coronary plaques' presence, extension, and vulnerable features.	*P* < 0.05

(Ji et al., 2018) [34]	Impact of sarcopenic obesity on 30-day mortality in critically ill patients with intra-abdominal sepsis	Investigate the association between sarcopenic obesity and 30-day mortality in patients with intra-abdominal sepsis.	2018	China	236	44 to 83	Retrospective cohort	Intra-abdominal sepsis	—	Sarcopenic obesity is an independent risk factor for 30-day mortality in critically ill patients with intra-abdominal sepsis.	*P* < 0.05

(Cho et al., 2019) [27]	The relationship between visceral adiposity and depressive symptoms in the general Korean population	Examine the association between clinical depressive symptoms and intra-abdominal fat.	2019	Korea	7238	Nondepressives (52.0 ± 8.7) Depressives (52.1 ± 9.8)	Cross-sectional	Depression	—	Depressive symptoms are associated with intra-abdominal fat and the proportion of total and visceral adipose areas in women.	*P* < 0.05

CRC: coronary heart disease (CHD); CT: computed tomography; SAT: subcutaneous adipose tissue; VAT: visceral adipose tissue; BMI: body mass index; NAFLD: nonalcoholic fatty liver disease; HDL-c: high-density lipoprotein cholesterol; LDL-c: low-density lipoprotein cholesterol; VLDL-c: very-low-density lipoprotein cholesterol.

## Data Availability

The article's data supporting this review are from previously reported studies and datasets, which have been cited. The processed data are available in Virtual Health Library (https://bvsalud.org/).
